# Cystic Echinococcosis in a Single Tertiary Care Center in Rome, Italy

**DOI:** 10.1155/2013/978146

**Published:** 2013-09-14

**Authors:** Linda Petrone, Gilda Cuzzi, Lidia Colace, Giuseppe Maria Ettorre, Elisa Busi-Rizzi, Vincenzo Schininà, Leopoldo Pucillo, Claudio Angeletti, Stefania Pane, Antonino Di Caro, Eugenio Bordi, Enrico Girardi, Edoardo Pozio, Angela Corpolongo, Antonella Teggi, Enrico Brunetti, Delia Goletti

**Affiliations:** ^1^Translational Research Unit, Department of Epidemiology and Preclinical Research, “L. Spallanzani” National Institute for Infectious Diseases (INMI), IRCCS, Via Portuense 292, 00149 Rome, Italy; ^2^Unit of Surgery and Transplantation, “Interaziendale” Department P.O.I.T. Polo Ospedaliero Interaziendale, San Camillo-INMI Lazzaro Spallanzani, Italy; ^3^Department of Radiology, INMI, Italy; ^4^Clinical Biochemistry and Pharmacology Laboratory, INMI, Italy; ^5^Department of Epidemiology and Preclinical Research, INMI, Italy; ^6^Microbiology Laboratory and Infectious Diseases Bio-Repository, INMI, Italy; ^7^Department of Infectious, Parasitic and Immunomediated Diseases, Istituto Superiore di Sanità, 00161 Rome, Italy; ^8^Clinical Department, INMI, Italy; ^9^Department of Infectious and Tropical Diseases, Sant'Andrea Hospital, Sapienza University of Rome, 00185 Rome, Italy; ^10^Department of Infectious Diseases, University of Pavia, IRCCS, S. Matteo Hospital Foundation, WHO Collaborating Centre for Clinical Management of Cystic Echinococcosis, 27100 Pavia, Italy

## Abstract

*Background*. Cystic echinococcosis (CE) is a chronic, clinically complex, and neglected disease. Its prevalence in Italy, a country of medium to high endemicity, remains poorly defined, as notification has long ceased to be mandatory. *Methods*. We set up a retrospective cohort study involving all CE patients followed at our institute between January 2005 and December 2012. Demographical and clinical features were recorded and analyzed. *Results*. CE was found in 28 patients (64.3%), mostly Italians from the central regions (50%), followed by subjects from the islands (33.3%) and Southern Italy (16.7%). Their median age was 45 years (IQR: 38.5–66.5), with Eastern Europeans being significantly younger (28 years, IQR: 19–39) than other patients (*P* ≤ 0.0001). A total of 149 cysts, mostly with hepatic localization (96%), were described. Based on the WHO classification, the cysts were mainly small (80.5%) and active (CE1 (73.8%); CE2 (7.4%)). Active cysts were more common in Eastern Europeans (85.7%) than Italians (66.7%). *Conclusion*. Our data confirm CE occurrence in Italy. We emphasize the importance to have a national CE registry, opportunely recently introduced. This is essential to assess CE prevalence in this country, implement appropriate control measures, and improve patient management.

## 1. Introduction 

Cystic echinococcosis (CE) is a zoonosis caused by the larval stage of the cestode *Echinococcus granulosus* whose adults and eggs are found in the small intestine of carnivores. CE is a neglected disease causing substantial morbidity and mortality in endemic areas [1, 2]. This helminthic disease has worldwide distribution, with endemic regions in many countries of the Mediterranean basin, Northern and Eastern Africa, Asia, South America, and Australia [3–8].

In Europe, published data suggest that CE prevalence is rather high in Turkey, Greece, Italy, Spain, and France [9]. Reemerging CE cases have been described in different European areas, for example, Wales and Spain, where high incidence rates of echinococcosis in dogs and new CE autochthonous cases in young people have recently been reported [10, 11].

With over 1,000 cases of surgery each year, Italy is considered a medium to high prevalence country for human CE [12]. CE is endemic in the regions of Southern Italy and the islands, mainly Sardinia, where CE has an average annual reported incidence of 4–8/100,000 inhabitants [13, 14]. Despite the increasing number of epidemiological reports [13–17], available information on CE distribution in Italy is still incomplete and insufficient to assess, even roughly, its epidemiology. Reporting human CE in Italy has long ceased to be mandatory (1991) and the ensuing disappearance of CE from the radar has eventually led to the institution of an Italian Registry of CE, which is almost complete at the time of this writing (http://www.iss.it/riec/). Contrary to reports by other authors from different European countries [18], human CE case numbers are underestimated [17] due to the peculiarities of CE transmission and difficulty in reading clinical presentation [19].

In 2011, the European community funded the project “Dissecting the Immunological Interplay between Poverty Related Diseases and Helminth infections: An African-European Research Initiative” (IDEA), with the aim of exploring the immunological interplay between poverty-related diseases and helminthic infections. Our preliminary work within this project aimed to evaluate the number of CE cases seen in our hospital. Therefore, we set up a retrospective study, considering all CE patients (in- and outpatients) cared for at the “Lazzaro Spallanzani” National Institute of Infectious Diseases (INMI) in Rome between January 2005 and December 2012. CE was also evaluated in relation to the status of HIV and tuberculosis (TB). In 2011, in Italy there were 3,461 new diagnoses of HIV infection, equivalent to an incidence rate of 5.8 per 100,000 residents, and 7.6 cases of TB per 100,000 residents were reported in 2008 [20, 21].

## 2. Materials and Methods

### 2.1. Epidemiological Survey

This observational retrospective cohort study was conducted at INMI, a hospital specializing in infectious diseases in Rome, Italy. More than 3,400 inpatients and 16,000 outpatients are cared for each year at INMI. We reviewed the data from CE patients who were followed at INMI between January 2005 and December 2012. Discharge diagnoses in the electronic records of the hospital were used to identify CE cases. Outpatients with evidence of echinococcal disease were notified by radiologists and clinicians. The study period was chosen based on the availability of data in the electronic database used by radiologists. The following information was collected from the clinical records or laboratory test results of patients with a confirmed diagnosis of CE: demographic data (age, sex, nationality, and country of birth), laboratory data (eosinophil count), diagnostic method (ultrasound (US) examination and parasitological serology), clinical data (symptoms), cyst details (number, localization, and size), treatment, and coinfections (HIV and active/past TB in particular), if any. Information was recorded in a database, and personal data were made anonymous, according to the current Italian regulations on privacy (L.675/96) [22].

### 2.2. CE Diagnosis

#### 2.2.1. US Examination

**Figure 1:**
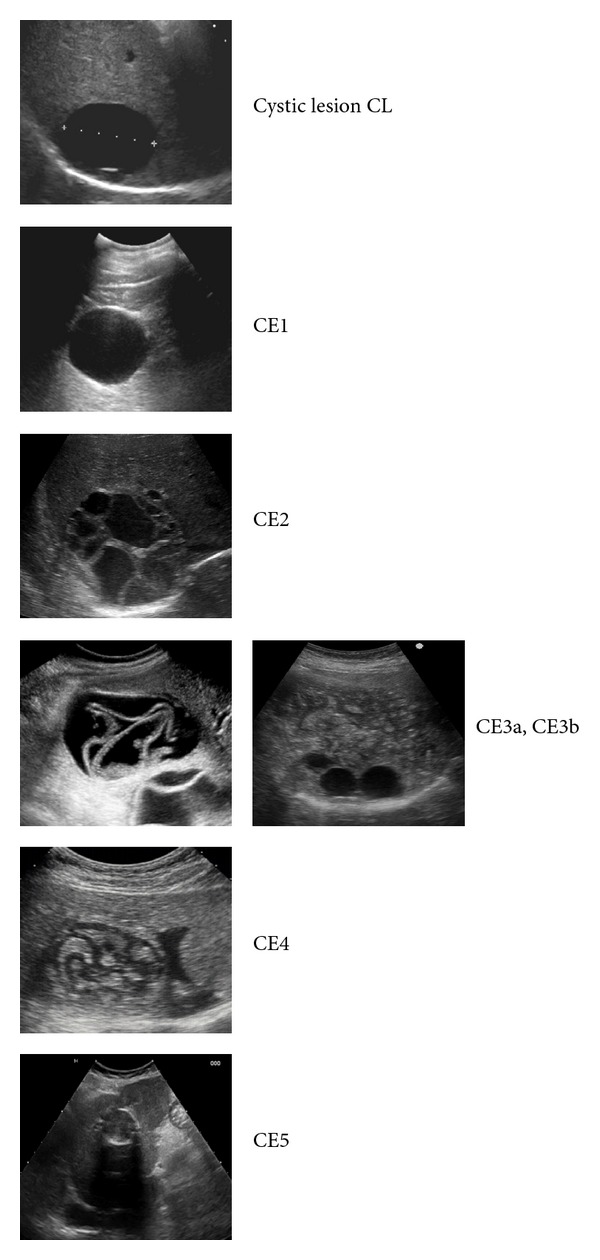
Summary of the cystic lesion (CL) and types of CE cysts described according to WHO ultrasound classification, showing active (CE1, CE2), transitional (CE3a, CE3b) and inactive (CE4, CE5) cyst types which follow the natural history of CE. CE: Cystic Echinococcosis.

Diagnosis of CE was made by US examination, using an iU22 machine (Philips, Eindhoven, The Netherlands) equipped with a broad-band curvilinear transducer (C5-1 Mhz). Each scan was video recorded and sent to a second, different observer to confirm or exclude CE and stage the cyst according to the World Health Organization (WHO) US classification. This includes CL, unilocular cystic lesion(s) with uniform anechoic content; CE1, unilocular cysts with uniform anechoic content and pathognomonic signs that include a visible cyst wall and “snowflake” signs; CE2, multivesicular, multiseptated cysts; CE3a, anechoic content with detachment of the laminated membrane from the cyst wall, visible as a floating membrane or as a “water lily sign,” and CE3b, predominantly solid with daughter cysts; CE4, heterogeneous hypoechoic or hyperechoic degenerative contents, without daughter cysts; and CE5 with a thick, calcified, arch-shaped wall which produces a cone-shaped shadow (Figure 1) [23].

#### 2.2.2. Serology

Serological diagnosis of *Echinococcus granulosus* at INMI was performed using IHA (Cellognost*-Echinococcosis, Marburg, Germany) and Western blot IgG (Euroimmun Labordiagnostika, Luebeck, Germany) kits. IHA results equal to or greater than 1 : 64 were considered positive when hemagglutinins against type O erythrocytes were absent. IHA-low positive titers of 1 : 32 to 1 : 128 could only be accepted as positive for echinococcosis if confirmed in conjunction with a second diagnostic method. *Echinococcus granulosus* Western blot IgG (Euroimmun Labordiagnostika, Luebeck, Germany) was used to evaluate bands detecting antibodies against *Echinococcus granulosus* antigens. These antigens were nonspecific antigens p39 (39 kDa), genus-specific but cross-reactive antigens with other Echinococcus species p24/26 (24–26 kDa), p16/18 (16–18 kDa), and also *Echinococcus granulosus* specific p7 (7 kDa) antigen. Manufacturer procedures were followed to evaluate the band patterns.

### 2.3. Active/Past TB Diagnosis

“Active TB” was defined by a positive culture for *M. tuberculosis* (Mtb) from sputa. “Past TB” was defined as culture-positive pulmonary TB in which completion of a 6-month course of treatment led to culture-negative sputa. “Radiological signs of probable past TB” included apical fibronodular lesions and/or apical calcifications.

### 2.4. HIV Serology

ARCHITECT HIV Ag/Ab Combo (Abbott Diagnostics, Wiesbaden, Germany) was used for HIV screening.

### 2.5. Statistical Analysis

Data were double entered and cross-checked, and statistical analyses were performed using SPSS v.19 for Windows (SPSS Italia SRL, Bologna, Italy). Gender, age, geographic origin, number and size of cysts, and treatment information in relationship to the WHO stage of the cysts were analyzed by univariate (Pearson's Chi-squared test for independence) statistical analysis. A *P* value of 0.05 was considered significant. For categorical variables, results were expressed as absolute numbers or percentages.

## 3. Results

### 3.1. Characteristics of the Population

**Figure 2:**
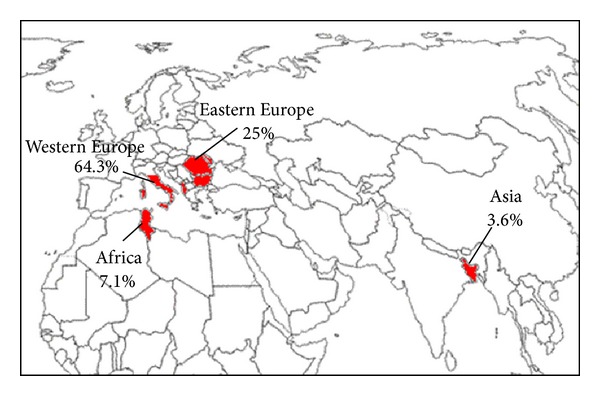
Choropleth map showing the origin of the total CE cases identified at INMI from 2005–2012.

We reviewed the data from 28 patients (18 inpatients with CE as a “discharge diagnosis” and 10 outpatients identified by the radiologists and clinicians as having CE) who were followed at INMI between January 2005 and December 2012. Demographic and clinical features are shown in Table 1. Data regarding the occupations of these patients were available in only 5 (17.9%). These subjects did not have a farming-related job, although all of them reported animal contacts (data not shown). Eighteen (64.3%) of the echinococcus-infected subjects were Italian. Among the Italians, 50% of the patients were born in central regions (Latium 7 (77.8%), Marche 1 (11.1%), and Abruzzo 1 (11.1%)). The remaining subjects came from Sardinia and Sicily (6 (33.3%)) and Southern Italy (3 (16.7%)). Seven (25%) subjects were from Eastern Europe, mainly Romania (4 (57.1%)). One (3.6%) patient came from Asia (Bangladesh) and 2 (7.1%) patients came from Africa (Tunisia) (Figure 2).

Most patients were females [16 (57.1%)]. The median age was 45 years (IQR: 38.5–66.5); the median age of the Italian patients (55.5 years, IQR: 41.5–72) was significantly higher than that of the non-Italians. In particular, Eastern European patients were significantly younger (28 years, IQR: 19–39) than Italian, Asian, and African patients (*P* ≤ 0.0001). Only five of these patients (17.9%) had been tested for HIV, and all of them were negative. Regarding TB status, 1 (3.6%) Italian patient had active pulmonary TB and 1 (3.6%) from Asia reported a past-cured pulmonary TB. Moreover, we evaluated the chest X-rays and lung images in 24/28 (85.7%) patients. Two out of the 24 (8.3%) patients showed radiological signs of probable past TB (apical fibronodular lesions and apical calcifications) although history of a previous disease was not reported in the clinical charts. Patients were not evaluated for latent TB infection (LTBI) by immune diagnostic tests.

Mild abdominal discomfort was the most common symptom reported (data not shown). Most patients (21 (75%)) were receiving albendazole and/or were scheduled for surgery at the time of study inclusion. Among them, 16 (57.2%) were treated with albendazole, 3 (10.7%) underwent surgical resection of the cysts, and 2 (7.1%) received a combination of medical and surgical treatments (Table 2). Information regarding previous treatment for CE was found in 7 patients (25%); in particular, 4 patients (14.3%) had received albendazole, 2 patients (7.1%) reported surgery, and 1 patient (3.6%) reported both.

### 3.2. Cystic Echinococcosis Serological Diagnosis

Serological diagnosis was performed using the IHA test and was confirmed by Western blot. Based on the manufacturer's instructions, IHA titers lower than 1 : 16 are scored negative for echinococcosis. In our survey, 10 patients (35.7%) had an IHA titer ranging from 1 : 16 to 1 : 128 and 15 patients (53.6%) had an IHA titer higher than 1 : 128 (Table 3). Only 3 subjects out of the total (10.7%) had an IHA titer lower than 1 : 16; however, 2/3 of these patients had a US diagnosis of CE which was later confirmed by surgery. Altogether, these data are consistent with CE diagnosis in all of the 28 analyzed cases.

### 3.3. Ultrasound Examination and Cyst Features

We performed a detailed analysis of the US reports available for all 28 CE infected subjects. 

As shown in Table 4, a total of 149 cysts were described in the US reports although the cysts/person ratio was low (cysts/person median: 1; IQR: 1-2), as reported in the literature. Most of the cysts were predictably located in the liver (143 (96%)).

We evaluated the chest X-ray for all the CE patients reported in the study with the exception of 4 (14.3%) who did not have lung images in the hospital records. However, in 2 out of 4 of these patients, we found a lung localization of cysts reported in their clinical chart. However, these cysts were not definitely identified as pulmonary CE cysts (data not shown). Among the 24 patients with lung images, 5 (20.8%) had pulmonary CE cysts (a total of 6 (4%) pulmonary cysts) and 19 (79.2%) did not show any sign of CE in the lungs. Moreover, one liver cyst eroded the diaphragm and extended into the lung (1 (0.7%)). Based on the WHO classification, 120 (80.5%) cysts were small, with a diameter smaller than 5 cm; 23 (15.5%) were medium sized, with a diameter ranging from 5 cm to 10 cm, and 6 (4%) cysts were large (>10 cm).

Most of the cysts were active, with 110 CE1 (73.8%) and 11 CE2 (7.4%), with far fewer CE4 and CE5 (2 (1.3%) and 16 (10.8%), resp.). There were 2 (1.3%) transitional CE3a cysts and 5 CE3b cysts (3.4%). Active cysts were more common among patients coming from Eastern Europe (85.7%) than in Italian patients (66.7%). Three cysts (2%) from 2 patients were undifferentiated and initially staged as CL. These cysts were later confirmed to be echinococcal, by surgery in 1 patient and by response to albendazole in the other patient. Cyst characteristics evaluated for each patient are shown in Table 5.

## 4. Discussion

Cystic echinococcosis is a chronic and neglected disease, and its pathogenesis and natural history are still poorly understood. CE, which occurs worldwide and causes significant losses in endemic areas, has a renewed importance in Europe, as reemerging cases have been documented [18]. The most affected regions in Western Europe are certain areas of Spain and Italy, in particular Southern Italy and Sardinia [9]. In Italy, mandatory notification of CE to the Italian Ministry of Health has been discontinued since 1991 (D. Min. San. 15.12.1991), and since then, the data on CE occurrence have only come from the summaries of the regional cases. Clinical presentation of CE in humans is complex, as echinococcal cysts may remain asymptomatic for years and then suddenly become complicated. In addition, the control of CE is difficult and requires cooperation between agricultural/veterinary services and medical authorities. Hence, the prevalence of human CE in Italy remains poorly defined.

This retrospective study shows that at our hospital CE was found in 28 patients over the course of 7 years. These patients had a US diagnosis of CE, complemented or not by positive serology. *Echinococcus granulosus*-infected subjects were mostly Italians coming from central regions, in particular from Latium (the region where the hospital is located), followed by subjects coming from the islands (Sardinia and Sicily) and Southern Italy.

CE is often an occupational disease, and farming and raising sheep seem to be risk factors [24]. Therefore, the prevalence of the disease is high on the islands and medium in the central and southern regions of Italy [16, 25] where farming and raising sheep are more frequent activities. In our study, the little data available regarding occupations and risk factors of CE subjects showed no correlation of CE with the occupations of our patients. Our results confirm the existence of CE in Italy, particularly in Latium, although it was impossible to determine whether CE infection was contracted locally or in regions with a higher endemicity.

Prevalence rates of human CE turned out to be higher than expected, also in regions considered nonendemic, such as Lombardy [17], indicating that there are patients from CE endemic areas residing in Lombardy. Moreover, it is unknown if risk factors associated with CE in humans are changing in different areas of Italy, and for this reason they should be specifically reassessed. Unfortunately, this reevaluation cannot be performed with the currently available information from the European Food Safety Authority (EFSA). In fact, no information on human CE cases and related aspects, such as occupation and other risks, has been recorded. This is why a national registry for CE with online data entry, modeled after the European Registry for Alveolar Echinococcosis [26], is needed. Several national surveillance systems for CE exist in European countries. Some of these are based on voluntary data entry (i.e., France, United Kingdom, and Belgium), while others are based on compulsory notification (i.e., Germany, Austria, etc.) [27], and data on CE cases can be reported by laboratories, physicians, or hospitals. Compulsory notification of CE and harmonization of the data entry system are essential instruments for surveilling the disease in humans. This would make collecting data on CE simpler, as it would eliminate the need to evaluate and integrate data from all regions, avoid duplication of data from patients who access several different health facilities over time, bypass conflict between inpatient and outpatient data, and make both clinical and epidemiological data accessible to clinicians, epidemiologists, and policymakers.

A low IHA titer was found in 35.7% of the CE subjects included in our study. In addition, 3 subjects (10.7%) had a negative IHA titer, although echinococcal cysts were documented and confirmed by surgery in 2 out of 3. This is in line with the literature on the poor performance of conventional serodiagnosis of CE. In fact, a key problem for immunodiagnosis is the high proportion of false-negative results (more than 25%) [28, 29] often due to the reduced amount of circulating antibodies. Inactive cysts, early CE1 cysts, or uncomplicated cysts in the lung often have negative serology results. Therefore, assessing CE cases by serology evaluation may lead to underestimating CE prevalence. As a consequence, WHO guidelines recommend a combination of imaging techniques, such as US and serology tests to ensure accurate diagnosis [30].

The majority of the subjects evaluated in this study had *Echinococcus granulosus* cysts in the liver. Most cysts were active (CE1, CE2) with a small diameter. This is an interesting finding, which points to active parasitic pressure in the areas where those patients acquired the infection [31]. In addition, 75% of the patients were undergoing albendazole treatment and/or were scheduled for surgery at the time of study inclusion.

Currently, it is under investigation whether helminths may negatively impact TB disease and HIV due to the rarity of available systematic studies [32, 33].

Also, among the CE patients evaluated in this study, TB was found as active TB or past TB in 1 patient, respectively, and “probable past TB” in 2, as defined by radiological signs; finally, only few patients were evaluated for HIV status. Therefore, it is difficult to analyze the impact of coinfections in CE. Recent papers suggest an association between CE and the immune suppression induced by HIV, since cases of severe disseminated disease have been reported with HIV coinfection [34, 35]. The fact that immune deficiency may have an impact on the clinical course and the epidemiology of CE may have important implications, mainly in HIV-endemic countries. However, only very few case reports of coinfection exist [36–38]. Therefore, larger cohort studies better addressing the effect of CE coinfection with HIV and/or TB disease and further investigation on the host-parasite interaction are needed.

The limitations of this study, such as the small number of subjects, the lack of information on occupational risks, and residency (rural versus urban areas) of the subjects prevent us from drawing conclusions. However, we provide a realistic picture of CE as seen in a hospital of infectious diseases in Italy and show that CE cases still occur in our country. This is especially important because there are no official European reports of human cases in Italy from 2004–2008 [39], a period partially covered by our study.

## 5. Conclusion

Information on CE prevalence in Italy is essential for implementing appropriate control measures and proper patient management. Our data confirm CE occurrence in Italy and the need to set up the prospective study within the IDEA initiative. A National Registry of Cystic Echinococcosis would be the key to fill the many gaps existing in the assessment of CE in Italy.

## Figures and Tables

**Table 1:** Demographic and clinical characteristics of the patients included in the study.

Origin	Patients	HIV		TB			Age (y)	Gender	Previous treatment (medical or surgical)	Treatment at INMI (medical or surgical)
	*N* (%)	HIV- *N*(%)	HIV unknown *N* (%)	Active TB *N* (%)	Past TB *N* (%)	Radiological signs of probable past TB *N* (%)*	Median (IQR)	F *N* (%)	*N* (%)	*N* (%)

Italy	18 (64.3)	3 (16.7)	15 (83.3)	1 (5.5)	—	1 (5.5)	55.5 (41.5–72)	10 (55.6)	3 (16.7)	14 (77.8)
Central	9 (50)	1 (33.3)	8 (53.3)	—	—	1 (100)	—	4 (40)	—	8 (57.1)
Southern	3 (16.7)	1 (33.3)	2 (13.4)	1 (100)	—		—	3 (30)	1 (33.3)	1 (7.2)
Islands	6 (33.3)	1 (33.3)	5 (33.3)	—	—		—	3 (30)	2 (66.7)	5 (35.7)
Eastern Europe	7 (25)	1 (14.3)	6 (85.7)	—	—		28 (19–39)	4 (57.1)	3 (42.8)	5 (71.4)
Romania	4 (57.1)	1 (100)	3 (50)	—	—		—	2 (50)	1 (33.3)	3 (60)
Other	3 (42.9)	—	3 (50)	—	—		—	2 (50)	2 (66.7)	2 (40)
Asia	1 (3.6)	1 (100)	—	—	1 (100)		49	—	—	—
Africa	2 (7.1)	—	2 (100)	—	—	1 (50)	43.5 (43-44)	2 (100)	1 (50)	2 (100)

Total	28 (100)	5 (17.9)	23 (82.1)	1 (3.6)	1 (3.6)	2 (8.3)	45 (38.5–66.5)	16 (57.1)	7 (25)	21 (75)

*N*: number; TB: tuberculosis; IQR: interquartile range; F: female; y: year. *radiological signs of “probable past TB” evaluated in 24 patients.

**Table 2:** Cystic echinococcosis treatment.

Origin	Patients *N* (%)	Previous treatment *N* (%)					Present treatment *N* (%)				
		None	Drug	Surgery	Drug and surgery	Unknown	None	Drug	Surgery	Drug and surgery	Unknown
Western Europe	18 (64.3)	14 (77.8)	2 (11.1)		1 (5.6)	1 (5.6)	2 (11.1)	10 (55.6)	2 (11.1)	2 (11.1)	2 (11.1)
Eastern Europe	7 (25)	3 (42.9)	1 (14.3)	2 (28.6)		1 (14.3)	1 (14.3)	4 (57.1)	1 (14.3)		1 (14.3)
Asia	1 (3.6)	1 (100)					1 (100)				
Africa	2 (7.1)	—	1 (50)			1 (50)		2 (100)			

Total	28 (100)	18 (64.3)	4 (14.3)	2 (7.1)	1 (3.6)	3 (10.7)	4 (14.3)	16 (57.2)	3 (10.7)	2 (7.1)	3 (10.7)

*N*: number.

**Table 3:** CE serology results of the patients included in the study.

Origin	Patients	Serology		
	*N* (%)	<1 : 16	1 : 16–1 : 128	>1 : 128
Italy	18 (64.3)	2 (11.1)	7 (38.9)	9 (50)
Eastern Europe	7 (25)	1 (14.3)	2 (28.6)	4 (57.1)
Asia	1 (3.6)	—	1 (100)	—
Africa	2 (7.1)	—	—	2 (100)

Total	28 (100)	3 (10.7)	10 (35.7)	15 (53.6)

*N*: number.

**Table 4:** Characteristics of the cysts in the CE patients.

Total cysts *N* (%)	Cysts/patient median (IQR)	Cyst size *N* (%)			Cyst stage *N* (%)							Cysts localization *N* (%)	
		<5 cm	5–10 cm	>10 cm	CL	CE1	CE2	CE3		CE4	CE5	Liver	Lung
								a	b				

149 (100)	1 (1-2)	120 (80.5)	23 (15.5)	6 (4)	3 (2)	110 (73.8)	11 (7.4)	2 (1.3)	5 (3.4)	2 (1.3)	16 (10.8)	143 (96)	6 (4)

*N*: number; IQR: interquartile range; CE: cystic echinococcosis.

**Table 5:** Serology and cyst details (number, stage, localization, and size).

PT	Gender	Origin	Age	Serology			Cysts (*N*)	Cyst stage	Cyst localization		Cyst diameter (cm)		
			(years)	<1 : 16	1 : 16–1 : 128	>1 : 128			liver	lung	<5	5–10	>10
PT1	F	Italy	49			X	1	C4	X		X		
PT2*	M	Romania	39			X	1	C5	X		X		
							1	C2	X			X	
PT3	F	Italy	34			X	2	C5	X			X	
PT4*	F	Italy	40			X	96	C1	X		X		
							1	C3a		X	X		
PT5	M	Italy	43			X	2	C2	X			X	
PT6*	M	Italy	78			X	1	C1		X		X	
							1	C5	X			X	
PT7*	M	Bulgaria	28			X	1	C2	X		X		
							1	C2	X		X		
							2	C3b	X		X	X	
PT8*	F	Italy	46		X		2	CL	X		X		
							1	C2	X			X	
PT9*	M	Italy	64		X		2	C1	X		X		
							1	C2	X			X	
PT10	M	Italy	82		X		1	C3b	X			X	
PT11*	F	Italy	72		X		1	C2	X		X		
							1	C3b	X				X
PT12	F	Moldavia	19	X			2	C1		X		X	
PT13	M	Romania	19		X		1	C1		X		X	
PT14	F	Romania	24		X		2	C1	X			X	
PT15	M	Bangladesh	49		X		3	C5	X		X		
PT16	F	Italy	46		X		1	C4	X		X		
PT17	F	Italy	77		X		2	C5	X		X		
PT18*	F	Tunisia	44			X	1	C5	X		X		
							1	C5	X			X	
PT19*	F	Italy	70			X	1	C5	X		X		
							1	C1	X		X		
PT20	F	Albania	39			X	1	CL	X				X
PT21*	F	Romania	38			X	2	C1	X		X		
							1	C3a	X			X	
PT22*	M	Italy	40		X		1	C1	X		X		
							1	C2	X			X	
PT23	F	Tunisia	43			X	1	C2	X			X	
PT24	M	Italy	69			X	1	C5	X				X
PT25	M	Italy	62			X	1	C2	X				X
PT26*	M	Italy	72			X	2	C5	X		X	X	
							1	C3b	X			X	
PT27	F	Italy	48	X			1	C5	X			X	
PT28*	F	Italy	25	X			1	C1	X				X
							1	C1		X			X

PT: patient; *cysts from the same patients; M: male; F: female; C: cyst.
